# Misdeployment of the metal stent during direct endoscopic ultrasound-guided hepaticogastrostomy without tract dilation

**DOI:** 10.1055/a-2769-5987

**Published:** 2026-04-20

**Authors:** Ryota Sagami, Yasuhisa Hiroshima, Fumihiko Asahina, Yoshifumi Azuma, Naoki Tamura, Kazunari Murakami, Kazuhiro Mizukami

**Affiliations:** 1 Department of Gastroenterology, Faculty of Medicine, Oita University, Oita, Japan; 2 Department of Advanced Gastrointestinal Cancer Medicine, Faculty of Medicine, Oita University, Oita, Japan

**Fig. 1:**
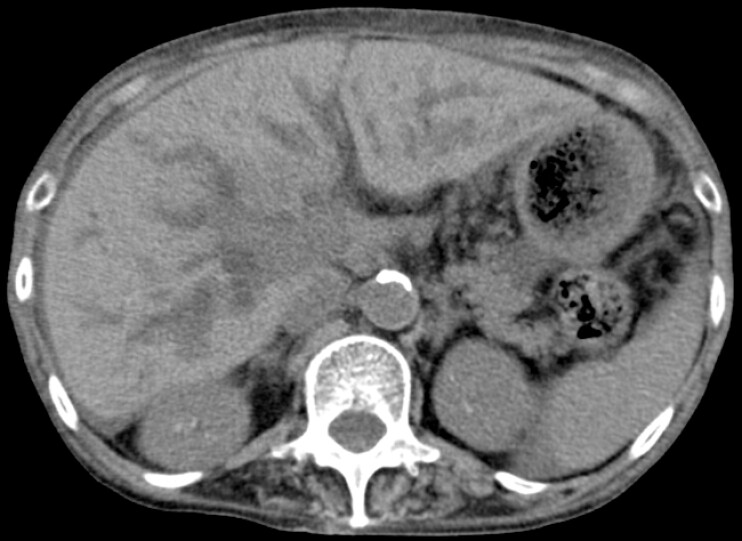
Computed tomography before endoscopic treatment. Dilated intrahepatic bile ducts due to recurrent pancreatic cancer after pancreaticoduodenectomy.

A 76-year-old woman was admitted for jaundice caused by recurrent pancreatic cancer after pancreaticoduodenectomy. Computed tomography (CT) revealed dilated intrahepatic bile ducts ( Fig. 1 ). Because of her poor respiratory condition, balloon enteroscopy-assisted endoscopy was avoided. Endoscopic ultrasound-guided hepaticogastrostomy (EUS-HGS) was performed using a partially covered self-expandable metal stent (SEMS) with a 5.9-Fr thin-delivery system (Hanaro-Benefit 8 mm/12 cm, Boston Scientific, Tokyo, Japan) without tract dilation, aiming for a shorter procedure time and to reduce the risk of bile leakage associated with dilation.

**Fig. 2:**
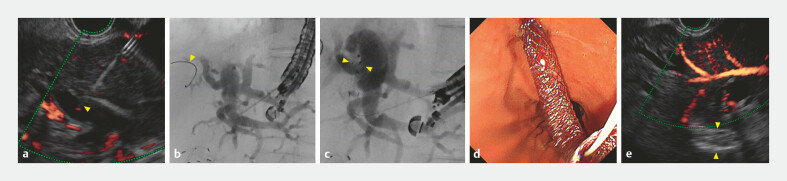
Images during the first endoscopic ultrasound-guided hepaticogastrostomy with stent misplacement. **a** Puncture of the B3 bile duct visualized on endoscopic ultrasound (yellow arrow head). **b** Advancement of the guidewire into the hilar bile duct with some unnatural movement under fluoroscopy (yellow arrow head). **c** Deployed stent visualized on fluoroscopy (yellow arrow heads). **d** No bile outflow observed from the stent lumen on the endoscopic view. **e** Upon EUS, the stent appeared to be located within a small peripheral bile duct or liver parenchyma beyond the target duct (yellow arrow heads).

**Fig. 3:**
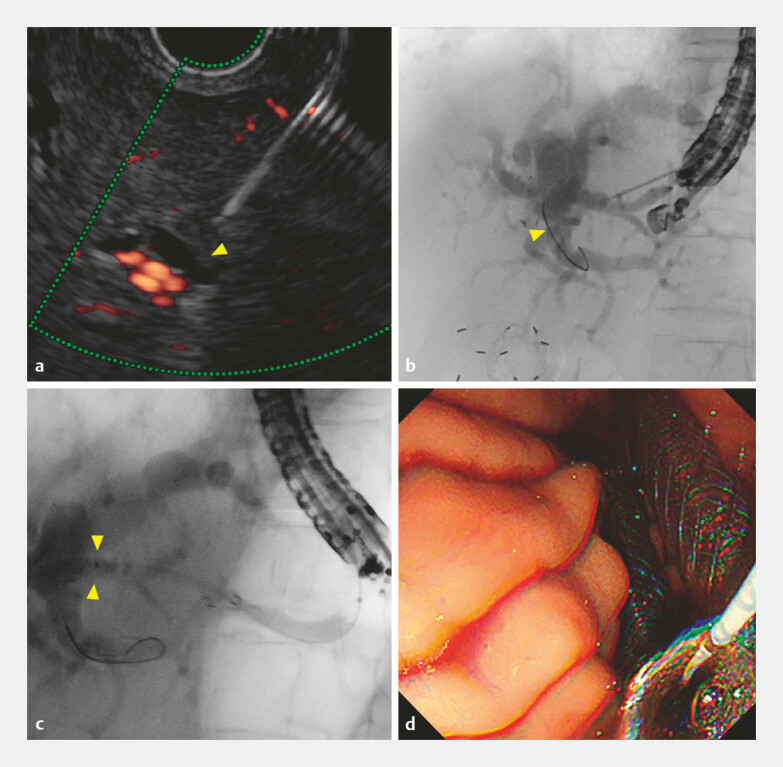
Images during the second successful endoscopic ultrasound-guided hepaticogastrostomy. **a** Puncture of another B3 bile duct visualized on endoscopic ultrasound (yellow arrow head). **b** The guidewire was inserted into the B2/3 confluence under fluoroscopy (yellow arrow head). **c** Placement of the rescue metal stent without dilation (yellow arrow heads). **d** Bile outflow confirmed endoscopically.

**Fig. 4:**
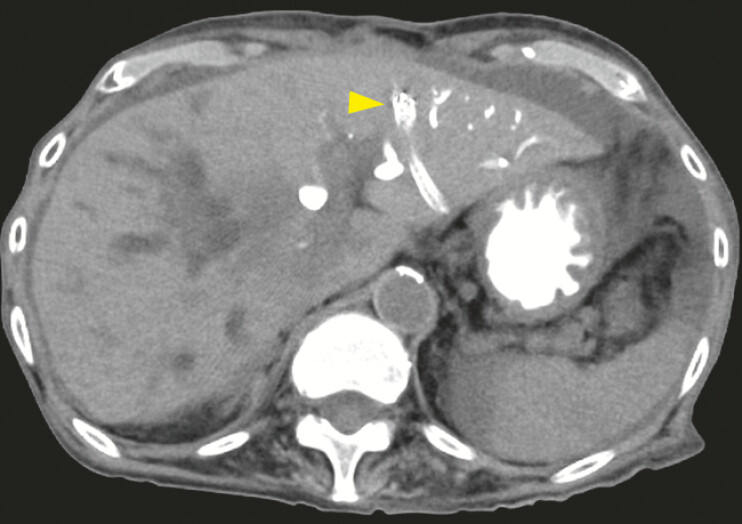
Computed tomography after endoscopic treatment. Misplacement of the initial metal stent into the hepatic parenchyma (yellow arrow head).

The B3 bile duct was punctured ( Fig. 2 **a** , Video 1 ). Contrast was injected ( Fig. 2 **b** ), and the guidewire was advanced into the hilar bile duct with some unnatural movement. The SEMS delivery system was then smoothly inserted, and the stent was deployed ( Fig. 2 **c** ); however, bile outflow was not observed ( Fig. 2 **d, e** ). Upon EUS, the SEMS appeared to be located within a small peripheral bile duct or liver parenchyma beyond the target duct ( Fig. 2 **e** ). Therefore, another B3 bile duct was re-punctured ( Fig. 3 **a** ), and a guidewire was inserted ( Fig. 3 **b** ) into B2/3 confluence. A rescue SEMS was then placed without dilation, and bile outflow was confirmed ( Fig. 3 **c, d** ). CT revealed the misplacement of the initial SEMS ( Fig. 4 ). The patient recovered uneventfully and was discharged after improvement of jaundice.

Endoscopic ultrasound-guided hepaticogastrostomy was performed without tract dilation using a 5.9-Fr delivery self-expandable metal stent. The B3 bile duct was punctured, and the guidewire was advanced into the hilar bile duct with unnatural movement, resulting in stent misplacement. A second B3 puncture enabled successful rescue stent placement with confirmed bile outflow and patient recovery.Video 1

Tract dilation may increase the risk of adverse events during EUS-HGS 1 . SEMS placement without dilation is a useful option to shorten and simplify the procedure 2 . In this case, misalignment between the scope and the guidewire view might have led to inadvertent migration of the guidewire into the hepatic parenchyma or Glisson’s sheath beyond the peripheral ducts after the initial puncture. This misdeployment might have been prevented by catheter insertion and cholangiography. When any unnatural movement of the guidewire is encountered, cholangiography using a catheter should be performed.

Endoscopy_UCTN_Code_CPL_1AL_2AD
